# Xanthogranulomatous Cystitis: A Challenging Imitator of Bladder Cancer

**DOI:** 10.1100/tsw.2010.139

**Published:** 2010-06-29

**Authors:** Sinan Ekici, Isin Dogan Ekici, Sevket Ruacan, Ahmet Midi

**Affiliations:** ^1^ Department of Urology, School of Medicine, Maltepe University, Istanbul, Turkey; ^2^ Department of Urology, School of Medicine, Yeditepe University, Istanbul, Turkey; ^3^ Department of Pathology, School of Medicine, Hacettepe University, Ankara, Turkey; ^4^ Department of Pathology, School of Medicine, Maltepe University, Istanbul, Turkey

**Keywords:** cystitis, histiocytes, xanthogranulomatous inflammation, urinary bladder

## Abstract

Xanthogranulomatous cystitis is a rare, benign, chronic inflammatory disease of the bladder, mimicking malignancy with unknown etiology. Herein, we report a 57-year-old man who presented with pollakiuria, nocturia, dysuria, left flank pain, and a palpable mass on the right lower abdomen. Computerized tomography demonstrated an obstructing 10-mm stone in the lower third of the left ureter and a 6-cm solid mass on the right at the anterolateral wall of the bladder. The mass presented local perivesical invasion at the anterolateral side. Cystouretroscopy revealed a mass protruding into the bladder cavity with edematous smooth surface. Frozen section analysis of the partial cystectomy specimen could not rule out malignancy. Therefore, radical cystoprostatectomy and ureterolithotomy were performed. Histologically, fibrosis, numerous plasma cells, eosinophils, and, immunohistochemically, CD68-positive epithelioid and foamy macrophages were detected. Localized prostatic adenocarcinoma was also found. The present case of xanthogranulomatous cystitis is the 23rd to be reported in the world literature.

## INTRODUCTION

Xanthogranulomatous inflammation is a type of chronic granulomatous inflammation histologically characterized by lipid-laden macrophages (xanthoma cells), multinucleated giant cells, cholesterol clefts, polymorphonuclear leukocytes, plasma cells and lymphocytes of polyclonal origin, fibrosis, and calcification. It is typically seen in the gall bladder and kidney, and is associated with obstruction due to stone disease with subsequent infection. It has been reported in many sites, including the colon, appendix, salivary gland, pancreas, brain, ovary, and endometrium, usually mimicking malignancy both clinically and macroscopically[1,2]. Xanthogranulomatous cystitis (XC) is an extremely rare, chronic inflammatory disease of the bladder. Herein, we report a unique case of XC mimicking solid bladder malignancy, accompanied with contralateral ureteral stone and prostatic adenocarcinoma.

## CASE REPORT

A 57-year-old man presented with pollakiuria, nocturia, dysuria, left flank pain, and a palpable mass on the right lower abdomen for 2 weeks. He had no significant past medical history related to inflammatory bowel disease, urinary tract infection, urinary stone, or any urologic intervention. He was a nonsmoker. Laboratory studies showed normal hematological and biochemical profiles. Prostate specific antigen level was 1.75 ng/ml. Urinalysis showed 15–19 red blood cells and 0–1 white blood cells per high-power field. Urine culture was negative. Computerized tomography (CT) of the abdomen and pelvis demonstrated grade 2 hydroureteronephrosis with an obstructing stone (10 mm) in the lower third of the left ureter, and a solid lesion (60 × 55 mm) with central necrosis in the right side of the anterolateral wall of the bladder. The mass was presented with local perivesical invasion at the anterolateral side (Fig. 1). Cystouretroscopy revealed a mass protruding into the bladder cavity with edematous, but smooth surface. The rest of the bladder mucosa was normal. CT-guided needle biopsy of the mass revealed fibrosis and necrosis. The patient underwent surgical exploration. The mass was in relation with the anterior abdominal wall and omentum with fibrotic adhesions. The bladder was released from the mass and partial cystectomy was performed initially. Frozen section analysis of the mass could not rule out malignancy because of fibrosis, necrosis, and presence of epithelioid and clear cells. Therefore, nerve-sparing radical cystoprostatectomy, orthotopic neobladder, and left ureterolithotomy were performed. Gross examination of the bladder revealed a yellow mass with areas of necrosis. There was no anatomical relation between the XC mass lesion and the urachus, and no urachal remnant was identified in the specimen. Histologically, fibrosis, numerous plasma cells, eosinophils, and histiocytes were detected (Figs. 2 and 3). Special immunohistochemical stains, such as Periodic Acid Schiff (PAS), Grocot’s Methenamine Silver (GMS), Gram stain, and Von Cossa, were applied on appropriate sections. PAS, GMS, and Gram stain showed no specific microorganism, neither bacteria nor fungi. Von Cossa stain showed no calcium deposition. Abundant CD68 (1:40, mouse monoclonal, Neomarkers, Westinghouse, U.S.) positive epithelioid and foamy macrophages were detected (Fig. 4). On histopathologic examination of the prostate, prostatic adenocarcinoma of Gleason score 6 (3+3) within the prostate involving less than 5% of the total prostate volume was also detected. The final histopathologic diagnosis was XC and prostatic adenocarcinoma. The patient was free of disease and continent 1 year after the operation.

## DISCUSSION

XC is a rare, chronic inflammatory disease that was first described by Wassilijew in 1932[3]. Following this case report, only 22 cases of XC have been reported in the entire literature[4,5,6,7]. The major clinical symptoms were lower abdominal mass, symptoms of cystitis, such as pollakiuria, urgency, and dysuria. The present case of XC is the 23rd to be reported in the world literature.

Xanthogranulomatous lesions develop on the basis of inflammation, tissue necrosis, and obstruction[2]. The etiology of XC has not been clarified since it is very rare. The development of XC may be influenced by various factors, including tumor bulk or dead tumor cells due to therapy, presence of an abundant amount of intravesical hemorrhage, immunological disorders, abnormal lipid metabolism, urethral stenosis, or pre-existing vascular compromise, such as atherosclerosis[1,4,5]. In reported cases, the etiology of XC was also proposed as urachal cyst or urachal remnant, or an abnormal host response to a malignancy that leads to chronic xanthogranulomatous inflammation[4,5]. However, only a few of them were associated with urothelial carcinoma or urachal carcinoma[3,4,5]. In the presented case, neither urachal cyst nor urachal remnant was found, but accompanying urolithiasis and prostatic adenocarcinoma were detected. However, since the lesion of XC is far from the left ureteral stone and the focus of prostatatic adenocarcinoma, none of these were considered to be etiologic factors for the development of XC. The only suspected cause might be a possible previous inflammatory process, with the finding of adhesions observed in the omentum and abdominal wall in this case. The other feature of the present case that should be mentioned is location of the mass. Most of the reported cases were localized mainly at the

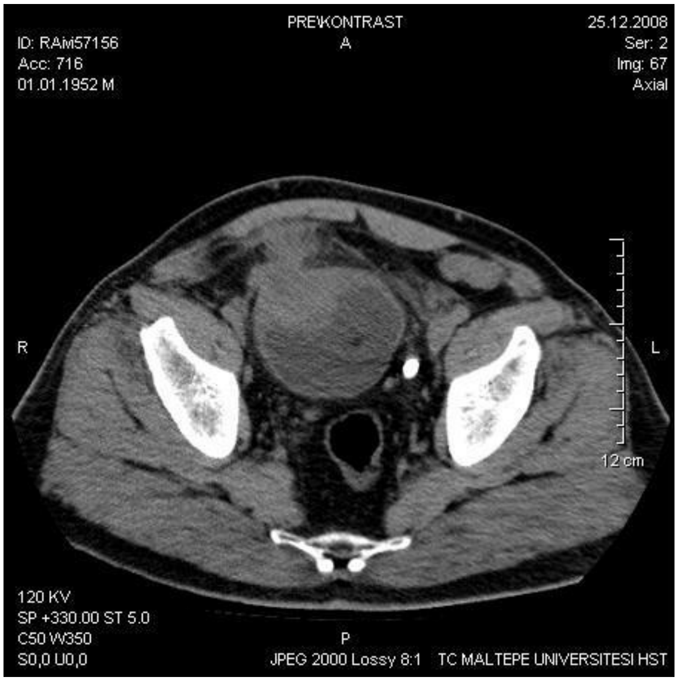

**FIGURE 1.** CT image without contrast; solid lesion (60 × 55 mm) with central necrosis in the right side of the anterolateral wall of the bladder was detected. The mass was presented with local perivesical invasion at the anterolateral side of the urinary bladder.

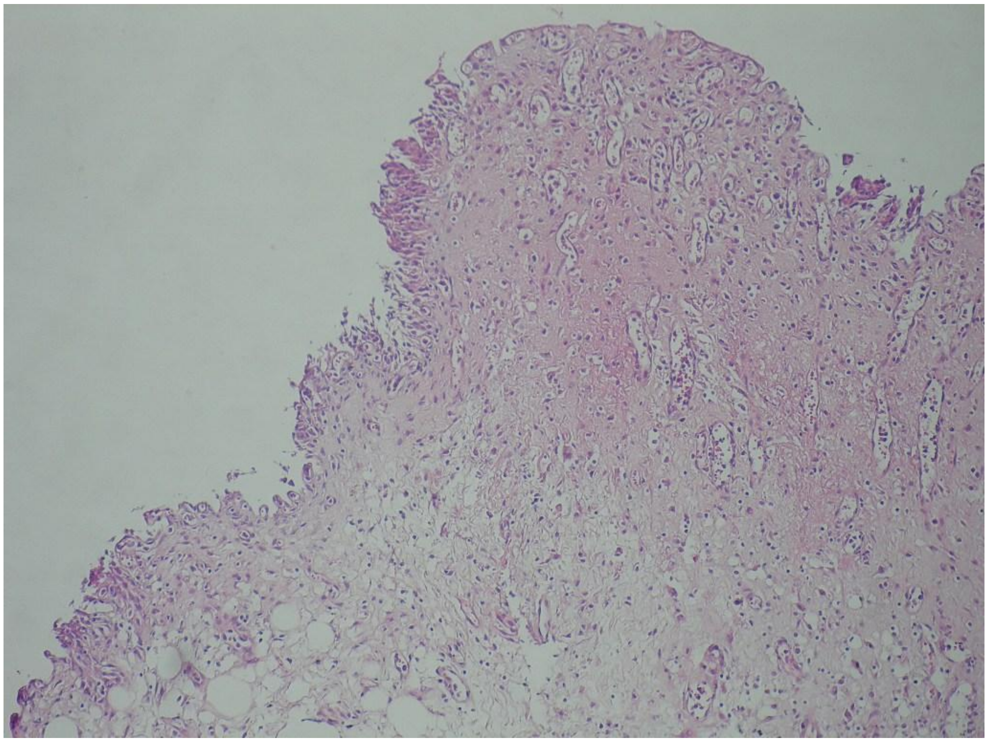

**FIGURE 2**. Edema, fibrosis, few plasma cells, eosinophil leukocytes, and histiocytes were seen in the superficial lamina propria (H&E, ×40).

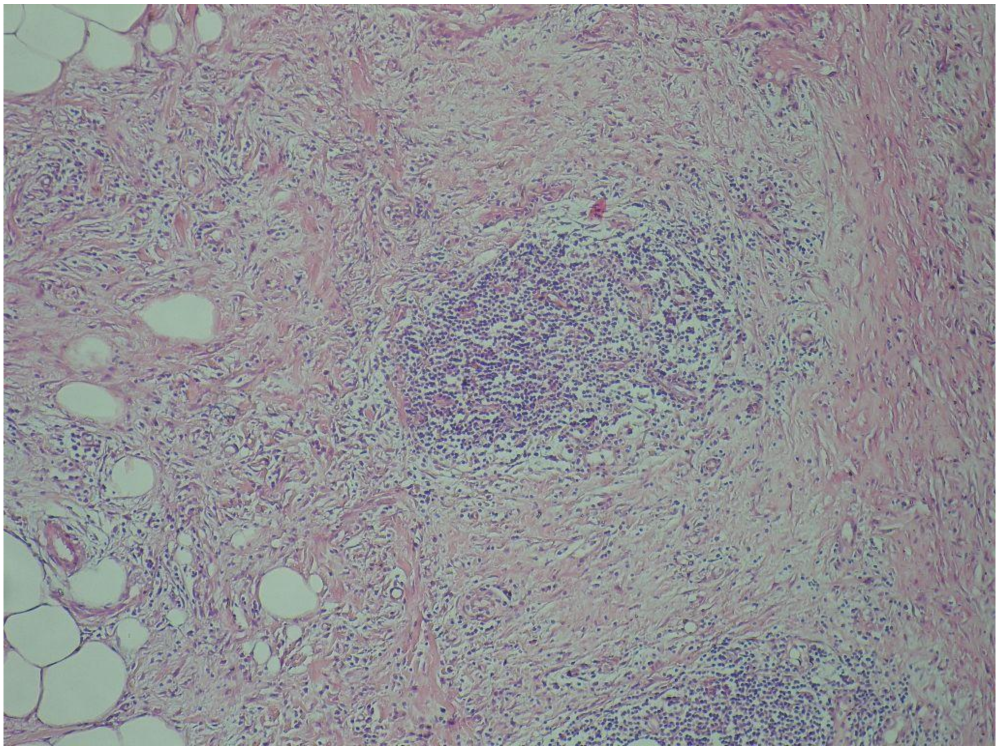

**FIGURE 3**. Dense fibrosis, numerous plasma cells, eosinophils, histiocytes, and lymphoid aggregates were detected in the deeper wall of urinary bladder (H&E, ×40).

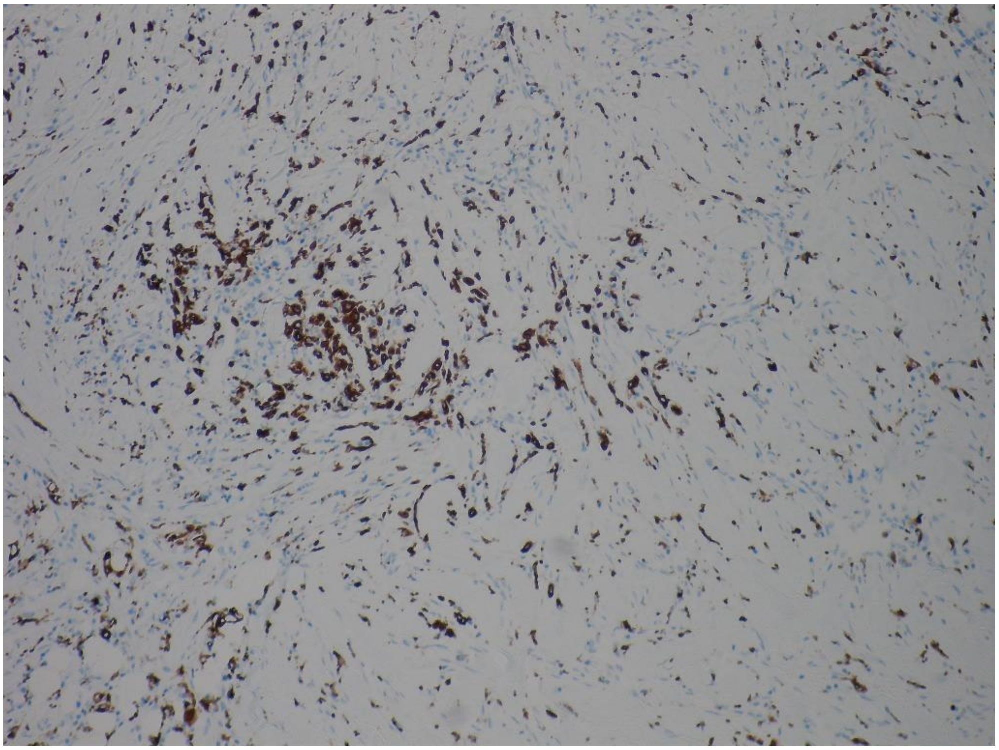

**FIGURE 4.** CD68-positive epithelioid and foamy macrophages were detected (×100).

dome of the bladder or near the dome[5]. However, in the present case, the mass was on the anterolateral wall of the bladder, which indicated absence of relation with the urachus.

Although a trial of long-term broad-spectrum antibiotics was suggested, conservative medical therapy is usually not effective and surgical resection is the gold-standard curative therapy[4,5,6, 7].

The irregular and necrotic appearance of XC may imitate carcinoma macroscopically[1,2,3,4,5]. Also, histologically, foamy histiocytes infiltrating the detrusor muscle may be misdiagnosed as sarcoma or a clear cell carcinoma, although the cytological and immunohistochemical features should simply resolve the problem[2]. Since XC imitates bladder cancer and both may coexist in the surgical specimen, keeping XC in mind in clinical and histopathological differential diagnosis of bladder mass lesions, and sampling of the entire surgical specimen during gross examination by pathologists, is essential[3].
